# Sociodemographic Indicators of Birth Rate in a Low Fertility Country–A Nationwide Study of 310 Finnish Municipalities Covering > 5,000,000 Inhabitants

**DOI:** 10.3389/fpubh.2021.643561

**Published:** 2021-04-23

**Authors:** Petteri Oura

**Affiliations:** ^1^Department of Forensic Medicine, University of Helsinki, Helsinki, Finland; ^2^Center for Life Course Health Research, Faculty of Medicine, University of Oulu, Oulu, Finland

**Keywords:** fertility, birth rate, general population studies, epidemiology, Finland

## Abstract

**Background and Aims:** Declining fertility is a key driver behind the rapid aging of populations worldwide. Finland has experienced a 25% decline in fertility from 2010 to date and ranks low even on the European and Nordic scales. This study aimed to address the association between sociodemographic indicators and birth rate (i.e., live births relative to total population) in Finland.

**Methods:** Open data on 310 Finnish municipalities were retrieved from the public database of Statistics Finland. Several sociodemographic subdimensions (population structure, education and income, location and living, divorces, car ownership rate, and crime rate), each converted to standard deviation units, were modeled against birth rate at the municipality level using generalized estimating equations.

**Results:** In this dataset, average annual birth rate was 8.8 per 1,000 individuals. Birth rate was positively associated with change in population size (rate ratio 1.06, 95% confidence interval 1.04−1.08), percentage of <15-year-olds (1.29, 1.22−1.36), percentage of individuals living in their birth municipality (1.05, 1.03−1.08), and percentage of foreign language speakers (1.02, 1.01−1.04). In contrast, birth rate was negatively associated with percentage of ≥65-year-olds (0.90, 0.85−0.96), percentage of unemployed individuals (0.98. 0.95−0.99), income (0.92, 0.89−0.96), and number of individuals living in the same household unit (0.94, 0.90−0.98).

**Conclusion:** The present findings are expected to advance the allocation of resources to areas and subpopulations that have high or low birth rate, and thus contribute to the development of a more family-friendly society. Future studies are encouraged to evaluate the sociodemographic indicators of birth rate in other low fertility countries, and to address the individual-level mechanisms behind the municipality-level associations identified in this study.

## Introduction

While the average 1950s woman had five children, the current global average is around 2.5 children per woman (1). Falling fertility has been suggested as the primary driver behind the rapid aging of populations worldwide, even overpowering the effect of reduced mortality (2, 3). It is evident that the global shift in population structure toward the older age groups imposes great challenges to public health (4, 5) and economy (2, 3).

There is regional variability in birth rate (i.e., live births relative to total population) and fertility rate (i.e., live births relative to women of reproductive age) across the globe (6). While two thirds of the world have fertility rates below the replacement level of 2.1 children per woman (i.e., Europe, Northern America, Australia and parts of the East Asia), there are also areas that clearly exceed this level (i.e., Africa and parts of the middle East) (2). Finland, after experiencing a dramatic 25% decline in fertility from 2010 onwards (7), ranks well below the European and Nordic averages with 1.4 children per woman (6). Although the underlying factors of this sudden decline remain somewhat unclear, the suggested public actions therein have gained news coverage in both national (8) and international media (9). It is nevertheless clear that Finland is currently verging on the threshold of lowest-low fertility (10).

The social determinants of health (11) include socioeconomic, societal, environmental and cultural factors which together constitute the circumstances one lives in. Further, these circumstances have an inevitable influence on the major decisions one makes throughout the lifespan, also during the reproductive years. Even though the drivers of declining fertility have been widely speculated in the global sense (1, 2), observational studies exploring the indicators of fertility at the population level have been relatively scarce. Previous studies have evaluated socioeconomic status (12–14), urban and rural living (15, 16), and foreign background (17, 18) relative to fertility. However, as these concepts are often intercorrelated, a multidimensional analysis is needed to reveal independent effects.

In the context of a low fertility country, this Finnish study aimed to identify sociodemographic predictors of birth rate at the municipality level. The fundamental aims were to advance the detection of areas and subpopulations that are inclined to high or low birth rate, and thus provide tools for an efficient resource allocation and development of a more family-friendly society.

## Materials and Methods

### Study Material

The material for this study was retrieved from the StatFin registry (19) which is an open, free-of-charge database accessible via the internet. StatFin is an umbrella database maintained by Finland's official statistical authority, Statistics Finland (20). Key statistics on Finland and its population are collected from various authorities and published at the national or regional level. Use of the material is allowed according to the CC BY 4.0 license (21). As the present study was retrospective, solely registry-based, and utilized a public dataset with no individual-level data, approval from an ethics committee was not necessary.

For this study, the StatFin database was queried for sociodemographic indicators available at the municipality level. The variables of interest were retrieved between October 2020 and January 2021, when the database followed Finland's current regional division into 310 municipalities. Data of all the 310 municipalities were retrieved from 2011 to 2018, i.e., the earliest and latest years when all the variables of interest were available, respectively. While Supplementary Table 1 provides links to precise variable descriptions, the following subsection presents an overview of the variables used in the study.

### Variables Used in the Study

#### Live Births and Population Structure

The data collections were based on Finnish citizens and foreigners legally residing in Finland for at least 12 months. The number of live births within a calendar year and total population size at the end of each year were obtained from the official Finnish population register maintained by the Digital and Population Data Services Agency (Digi- ja väestötietovirasto). Change in population size was calculated as the difference between two consequent years in percentages, with positive and negative percentages indicating increase and decrease in population, respectively. Population structure was further estimated by calculating the percentages of females, <15 and ≥65-year-olds relative to the total population of each municipality, on the basis of the population register. Here, the general birth rate was chosen as the outcome instead of fertility rate as the study aimed to identify sociodemographic predictors of birth count across Finnish municipalities, in contrast to studying the predictors of female fertility.

#### Education and Income

Data on completed educational qualifications and degrees in Finland and abroad were obtained from several databases which are mainly updated by educational institutions. Individuals are classified according to highest completed education into primary, secondary (i.e., high school leading to matriculation examination; vocational school; specialist vocational qualifications) and tertiary levels (i.e., polytechnic; university). Here, low education was estimated by calculating the percentage of individuals with only primary education relative to all ≥15-year-olds.

Employment and occupational statistics are constructed on the basis of several data sources such as Finnish Tax Administration (Verohallinto), Social Insurance Institution of Finland (Kela), employment registers, and student and conscript registers. For this study, municipalities' unemployment rates were calculated as the percentage of unemployed relative to the labor force (i.e., individuals who are either employed or unemployed, excluding students, conscripts and retired individuals). Data on income were obtained from the registry of Finnish Tax Administration. The registry includes all individuals who receive taxable incomes within a year. Here, the median annual gross income (in euros) of each municipality was used in the analyses.

#### Location and Living

The location of individuals, i.e. address, was obtained from the population register. Population density (individuals per km^2^) of each municipality was calculated as total population size divided by land area. Land areas were obtained from the official records of National Land Survey of Finland (Maanmittauslaitos). Division between individuals living urban and rural was made according to urban-rural classification of Finnish Environment Institute (Suomen ympäristökeskus). The classification is geographical information system-based and allocates 250 × 250-meter map grids as urban areas (i.e., agglomerations with >15,000 residents and their peri-urban area) or rural areas (i.e., not identified as urban).

Housing conditions were estimated on the basis of records from the population register and Finnish Tax Administration. Data on buildings and households are collected and reported by municipal building inspection authorities as part of building permit processes. A household unit is defined as a building which has its own entrance, encompasses at least one room and a cooking area, and is intended for permanent habitation. The number of residents in a household unit was determined by permanent addresses. The percentage of overcrowded household units (i.e., units in which the number of residents exceed the number of rooms excluding kitchen) was calculated relative to all household units. The percentage of individuals whose current municipality of recidence was the same as their municipality of birth was also calculated.

#### Other Sociodemographic Indicators

The percentage of foreign language speakers (i.e., other than Finnish, Swedish and Sami) was calculated using records from the official population register. Divorce rate (i.e., number of granted divorces relative to total population) was based on data reported by courts of law to the population register. Car ownership rate was calculated as the total number of registered passenger cars relative to total population. Car registration data were obtained from the traffic affairs register of Finnish Transport and Communications Agency (Liikenne- ja viestintävirasto). Finally, crime rate (i.e., total number of offenses registered by authorities relative to total population) was constructed using data from the Ministry of Interior's police information system.

### Statistical Analysis

The characteristics of the municipalities were first explored using descriptive statistics. Means with standard deviations or medians with interquartile ranges were presented, depending on the distribution of data. Characteristics were tabulated according to rough population size tertiles (<4,000, 4,000−9,999, ≥10,000), and differences between the tertiles were analyzed using one-way analysis of variance or Kruskal–Wallis test, depending on the distribution of data.

After considering previous literature (22), the association between municipality-level sociodemographic variables and birth rate was analyzed using generalized estimating equations (GEE). The negative binomial model with log link was applied, with the number of live births as the outcome, and total population size as the offset variable. All predictors were standardized (i.e., mean and standard deviation set to 0 and 1, respectively) before implementing them into the GEE procedure, in order to allow interpretation of regression coefficients (and exponentiated regression coefficients, i.e., rate ratios, RRs) in standard deviation units. Both univariate and multivariable models were constructed. Annual data were considered to be nested within municipalities. After comparing working correlation matrix structures by means of the Quasi-likelihood under Independence Model Criterion (QIC) and the Corrected Quasi-likelihood under Independence Model Criterion (QICC), the ‘exchangeable' correlation structure was selected as it provided the best fit in this dataset. All data were available for each municipality, with no missing data. RRs, their 95% confidence intervals (CIs), and the corresponding *P*-values were extracted from the GEE output.

As a sensitivity analysis, the models were re-run after excluding the 1 and 99% percentiles of each variable and including interaction terms between time and each predictor in the models. These approaches did not essentially alter the results of the initial models. Statistical analysis was performed using SPSS Statistics version 26 (IBM, Armonk, NY, USA). *P*-values < 0.05 were considered statistically significant.

## Results

The analysis was based on 310 Finnish municipalities with population sizes ranging from 91 to 648,042. Characteristics of the municipalities are presented in Table 1. Approximately 50% of the population were female, 16% were <15 years old, and 25% were ≥65 years old. Mean unemployment rate was 12%, and secondary or tertiary education was lacking from 35% of the population. On average 8.8 live births occurred annually per 1,000 individuals. Small, medium and large municipalities showed differences in all characteristics except for unemployment rate.

**Table 1:** Characteristics of the Finnish municipalities in 2011–2018.

**Indicator**	**All municipalities** **(*n* = 310)**	**Municipalities by population size**			
		** <4,000** **(*n* = 108)**	**4,000−9,999** **(*n* = 99)**	**≥10,000** **(*n* = 103)**	***P*-value**
Population structure					
Annual birth rate (per 1,000 individuals)	8.8 (3.1)	7.7 (3.5)	8.9 (3.1)	9.8 (2.2)	** <0.001**
Annual change in population size (%)	−0.6 (1.3)	−1.1 (1.4)	−0.7 (1.1)	+0.0 (0.9)	** <0.001**
Females (%)	49.5 (1.3)	48.6 (1.4)	49.5 (0.9)	50.5 (0.8)	** <0.001**
<15-year-olds (%)	15.9 (4.0)	14.3 (3.9)	16.5 (4.3)	17.1 (3.2)	** <0.001**
≥65-year-olds (%)	24.9 (6.1)	28.4 (5.5)	24.9 (5.9)	21.2 (4.5)	** <0.001**
Education and income					
Low education (%)	34.9 (5.7)	38.8 (4.7)	34.8 (4.6)	30.6 (4.4)	** <0.001**
Unemployment (%)	11.9 (4.4)	11.9 (5.0)	12.0 (4.4)	11.8 (3.8)	0.882
Annual income (eur)	21 668 (3374)	19 946 (2914)	21 595 (3270)	23 615 (2868)	** <0.001**
Location and living					
Population density (per km^2^)^*^	10.7 (5.2−25.1)	5.2 (3.4−9.0)	10.0 (5.7−15.8)	33.3 (19.1−84.6)	** <0.001**
Individuals living in rural area (%)^*^	98.8 (30.3−99.3)	99.1 (98.7−99.4)	98.9 (91.7−99.3)	19.1 (4.2−97.6)	** <0.001**
Individuals per household unit	2.1 (0.2)	2.1 (0.2)	2.2 (0.3)	2.1 (0.2)	** <0.001**
Overcrowded household units (%)	8.8 (2.1)	8.9 (2.3)	9.0 (2.3)	8.3 (1.5)	** <0.001**
Individuals living in municipality of birth (%)	50.5 (12.1)	53.7 (9.2)	51.2 (13.2)	46.1 (12.6)	** <0.001**
Other indicators					
Foreign language speakers (%)^*^	2.0 (1.2−3.3)	1.6 (1.0−3.0)	1.7 (1.2−2.5)	2.7 (1.9−4.2)	** <0.001**
Divorce rate (per 1000 individuals)	2.0 (0.9)	1.7 (1.1)	1.9 (0.7)	2.4 (0.5)	** <0.001**
Car ownership rate (per individual)^*^	0.65 (0.61−0.70)	0.69 (0.64−0.74)	0.66 (0.62−0.70)	0.61 (0.57−0.65)	** <0.001**
Crime rate (per individual)^*^	0.11 (0.08−0.16)	0.08 (0.06−0.12)	0.12 (0.09−0.19)	0.14 (0.11−0.17)	** <0.001**

*Values are means with standard deviations unless otherwise indicated.*

* *Median ± interquartile range. Bold values signifies statistical significance*.

Initial univariate and final multivariable GEE models for the association between sociodemographic indicators and birth rate are presented in Table 2. Partial multivariable models, to which predictors were added block by block, are presented in Supplementary Table 2. RRs of the predictors are interpreted in standard deviation units. Intercorrelations of the predictor variables are presented in Supplementary Table 3.

**Table 2:** Association between sociodemographic indicators and birth rate in Finland in 2011–2018.

**Indicator**	**Univariate models**			**Final multivariable model**		
	**RR**	**95% CI**	***P*-value**	**RR**	**95% CI**	***P*-value**
Population structure						
Total population size	**1.06**	**1.02; 1.09**	**0.002**	1.00	0.98; 1.03	0.753
Annual change in population size (%)	**1.09**	**1.08; 1.11**	** <0.001**	**1.06**	**1.04; 1.08**	** <0.001**
Females (%)	1.02	0.98; 1.06	0.284	1.01	0.99; 1.04	0.346
<15-year-olds (%)	**1.30**	**1.27; 1.33**	** <0.001**	**1.29**	**1.22; 1.36**	** <0.001**
≥65-year-olds (%)	**0.78**	**0.76; 0.80**	** <0.001**	**0.90**	**0.85; 0.96**	** <0.001**
Education and income						
Low education (%)	**0.87**	**0.84; 0.89**	** <0.001**	0.97	0.94; 1.01	0.125
Unemployment (%)	**0.94**	**0.91; 0.96**	** <0.001**	**0.98**	**0.95; 0.99**	**0.044**
Median annual income (eur)	**1.14**	**1.11; 1.17**	** <0.001**	**0.92**	**0.89; 0.96**	** <0.001**
Location and living						
Population density (per km^2^)	**1.03**	**1.01; 1.06**	**0.008**	1.01	0.99; 1.03	0.495
Individuals living in rural area (%)	**0.92**	**0.90; 0.95**	** <0.001**	1.01	0.99; 1.03	0.478
Individuals per household unit	**1.22**	**1.19; 1.25**	** <0.001**	**0.94**	**0.90; 0.98**	**0.004**
Overcrowded household units (%)	**1.14**	**1.11; 1.17**	** <0.001**	0.99	0.96; 1.02	0.488
Individuals living in municipality of birth (%)	**0.93**	**0.90; 0.96**	** <0.001**	**1.05**	**1.03; 1.08**	** <0.001**
Other indicators						
Foreign language speakers (%)	1.02	0.99; 1.05	0.103	**1.02**	**1.01; 1.04**	**0.019**
Divorce rate	1.00	0.99; 1.01	0.570	1.00	0.99; 1.02	0.807
Car ownership rate	**0.95**	**0.91; 0.98**	**0.004**	1.02	0.99; 1.04	0.096
Crime rate	1.00	0.98; 1.02	0.995	1.00	0.99; 1.01	0.872

*Predictors were standardized before fitting the GEE models, making results harmonized into standard deviation units. CI, Confidence interval; GEE, Generalized estimating equations; RR, Rate ratio. Bold values signifies statistical significance*.

According to the final multivariable GEE model, birth rate was positively associated with change in population size (RR 1.06, 95% CI 1.04−1.08), percentage of <15-year-olds (1.29, 1.22−1.36), percentage of individuals living in their birth municipality (1.05, 1.03−1.08), and percentage of foreign language speakers (1.02, 1.01−1.04). In contrast, birth rate was negatively associated with percentage of ≥65-year-olds (0.90, 0.85−0.96), percentage of unemployed individuals (0.98. 0.95−0.99), income (0.92, 0.89−0.96), and number of individuals living in the same household unit (0.94, 0.90−0.98). The remaining indicators (i.e., total population size, percentage of females, low education, population density, urban-to-rural-ratio, household overcrowdedness, divorce rate, car ownership rate, and crime rate) showed no statistically significant independent association with birth rate.

## Discussion

This nationwide study utilized open data from 310 Finnish municipalities in an attempt to reveal municipality-level sociodemographic indicators of birth rate. Indeed, the analysis identified several independent indicators of higher birth rate (positive change in population size and higher percentages of <15-year-olds, individuals living in their birth municipality, and foreign language speakers) as well as lower birth rate (higher percentage of ≥65-year-olds and unemployed individuals, higher median income, and higher number of individuals living in the same household). In addition to confirming associations that were previously known, this study also revealed novel information on the set of indicators that have value in estimating birth rate in a low fertility country.

Population structure and birth rate are at a constant interplay. It is clear that birth rate is one of the key factors contributing to the increase or decrease in a municipality's population size, and that municipalities with a higher proportion of fertile-aged individuals also tend to have a higher number of young families (2). These concepts were clearly reflected in the present associations of population growth, <15- and ≥65-year-olds with birth rate. In contrast, birth rate was not independently associated with sex distribution, population size, population density, or urban-to-rural ratio. The initial univariate associations of these indicators attenuated after the inclusion of the remaining predictors in the final multivariable model. Thus, although the Finnish municipalities have great variability in population size, density, and urban-rural-division, these characteristics seem to explain only a small fraction in birth rate variability between municipalities. The present findings are somewhat contrary to those of previous Finnish (15) and Nordic studies (16) which concluded that fertility is highest in small towns and rural areas and lowest in larger settlements. In addition to methodological discrepancy between the studies, the differing results may also reflect a temporal shift in the association over the previous decade.

A novel finding of this study was the positive association between individuals currently living in their municipality of birth and birth rate. It may be that individuals who do not need to relocate are able to settle down and consider starting a family earlier. Alternatively, the association may reflect the possibility of certain municipalities having low move-out rates due to, e.g., central location which simultaneously attracts young families to move in. A previous Austrian study (23) found that childbearing typically induces residential relocations, though mostly within a labor-market area. Importantly, the present finding was independent of all the other indicators assessed in the models, including geographical and socioeconomic factors. Future studies are encouraged to unravel the underlying mechanisms of this association further.

Of direct socioeconomic indicators, unemployment and median income had an independent association with birth rate, while low education did not. A closer look at RRs indicates, firstly, that within the pool of socioeconomic indicators, mean income exceeds both low education and unemployment rates in predictive value at the municipality level. Secondly, the findings suggest that areas of higher wealth have lower reproduction rates and vice versa. This phenomenon has been widely recognized and discussed (1, 12–14), with education- or career-orientedness and older age at reproductive onset constituting some of the main underlying mechanisms. Less traditional approaches to gender roles may also have a role in the equation (24, 25).

Foreign language speakers were associated with higher birth rate at the municipality level. This finding is likely to be mediated by foreign background and cultural traditions in starting a family. Recent papers (17, 18) reported great heterogeneity in fertility among descendants of immigrants in Europe, an observation which underlines the diversity of individuals and families with foreign background.

The number of individuals in a household unit, a proxy for average family size, was negatively associated with birth rate in the multivariable models. This may be explained by residual confounding, as the percentage of <15-year-olds had a strong positive association with birth rate in the dataset. Alternatively, large family size may indicate lower desire for a new child. Correspondingly, household overcrowdedness was not associated with birth rate in the full model; its potential effect is likely to be mediated by geographical factors (i.e., population density), socioeconomic status (i.e., poor households), and age distribution (i.e., large young families).

Divorce rate had no association with birth rate in this dataset. Despite a Nordic report (26) which concluded that marriage and childbearing are generally perceived as two linked concepts, the present data did not support the idea of an interplay between divorce rate and birth rate. Car ownership rate, proxying the ability to travel, was overpowered by the other indicators, but showed a trend of borderline significance toward higher birth rate. Crime rate showed no association with birth rate in any of the models, indicating that reproduction and criminal activity are separate phenomena at the municipality level.

This study had several strengths. First, the dataset was comprised of nationwide data from a country with a generally low birth rate. The datased covered all municipalities and included data from 2011 to 2018 for maximal representativeness. There were no missing data. Second, all data were based on official statistics recorded and distributed by Statistics Finland. Thus, the data were reliable and accompanied by detailed descriptions regarding the construction of each variable. Third, the present analysis was based on a wide range of sociodemographic indicators including population structure, education and income, location and living, divorces, car ownership rate, and crime rate. The analysis was performed using GEE which accounted for the internal correlations within the data. A multivariable approach was considered highly valuable in order to reveal the independent effect of each predictor relative to the outcome. Finally, all data used in this study are public and available for further scrutiny under the CC BY 4.0 license.

There were also several limitations to this study. First, only municipal-level data on birth rate and the socioeconomic parameters were available. However, similar apporaches have been used elsewhere (27), and the present approach led to the revelation of a clear set of indicators which predict birth rate. While the present results need to be interpreted only at the municipal level, they give clear implications of sociodemographic factors that are associated with high and low birth rate. Second, as the data used in this study were public and already collected before the initiation of this study, the methodology and range of variables available at the municipality level could not be influenced. In spite of the relatively large selection of sociodemographic variables available at the municipality level, several important population indicators could not be addressed. For example, health indicators such as smoking, obesity and physical activity were not available. Finally, the study addressed Finland only, and does not provide direct evidence regarding other countries or nationalities. However, while future studies are encouraged to explore the predictors of birth rate in other low fertility countries, the present findings are expected to possess information value also outside the Finnish context. Future studies are also encouraged to address fertility rate as the outcome instead of the general birth rate.

The public health implications of this study are manifold. In a low fertility country such as Finland, the study of birth rate indicators is of high importance as they may advance the detection of areas and subpopulations that are inclined to high or low birth rate. Evidence-based data on birth rate indicators may be used to guide resource allocation in order to develop a more family-friendly society and, ultimately, influence fertility. Firstly, efforts to reduce unemployment and poverty seem advisable also from the viewpoint of fertility. Secondly, resources could be directed to education- and career-oriented individuals (proxied by high income) to better facilitate parenthood via, e.g., parental leaves, daycare services or other means of municipal support. Thirdly, it seems advisable for municipalities to attract young citizens to reside in the municipality instead of relocating. Fourthly, the present findings underline the importance of robust and well-organized maternity and child health clinics in municipalities that meet the characteristics associated with higher birth rate. In particular, municipalities should aim to ensure that all services are readily available and accessible for foreign-language speakers. Lastly, despite these remarks, the author wishes to emphasize that an individual's voluntary choice not to have children should always be respected.

## Conclusion

This Finnish nationwide study identified municipality-level sociodemographic indicators of higher birth rate (positive change in population size and higher percentages of <15-year-olds, individuals living in their birth municipality, and foreign language speakers) and lower birth rate (higher percentage of ≥65-year-olds and unemployed individuals, higher median income, and higher number of individuals living in the same household). In a low fertility country, the present findings are expected to advance the allocation of resources to areas and subpopulations that have high or low birth rate, and thus contribute to the development of a more family-friendly society. Future studies are encouraged to evaluate the sociodemographic indicators of birth rate in other low fertility countries, and to address the individual-level mechanisms behind the municipality-level associations identified in this study.

## Data Availability Statement

Publicly available datasets were analyzed in this study. This data can be found here: https://www.stat.fi/tup/tilastotietokannat/index_en.html (StatFin database, Statistics Finland).

## Ethics Statement

Ethical review and approval was not required for the study on human participants in accordance with the local legislation and institutional requirements. Written informed consent from the participants' legal guardian/next of kin was not required to participate in this study in accordance with the national legislation and the institutional requirements.

## Author Contributions

PO is the sole author of the manuscript and worked on the conception of the project, data curation, analysis and interpretation of the data, and writing the manuscript.

## Conflict of Interest

The author declares that the research was conducted in the absence of any commercial or financial relationships that could be construed as a potential conflict of interest.
