# Supplementary material for: Sociodemographic Indicators of Birth Rate in a Low Fertility Country–A Nationwide Study of 310 Finnish Municipalities Covering > 5,000,000 Inhabitants
Source: Front Public Health. 2021 Apr 23;9:643561. doi: 10.3389/fpubh.2021.643561 (PMC8104080; doi:10.3389/fpubh.2021.643561)
Supplement: Supplementary file 1 [file Table_1.DOCX]

**Supplementary Table 1**. Links to detailed variable descriptions.

| **Variable** | **Link to variable description** | **Reference** |
| --- | --- | --- |
| Live births | http://www.stat.fi/til/synt/meta_en.html  http://www.stat.fi/til/synt/kas_en.html | Official Statistics of Finland (OSF): Births [e-publication]. ISSN=1798-2413. Helsinki: Statistics Finland. |
| Total population size | http://www.stat.fi/til/vaerak/meta_en.html  http://www.stat.fi/til/vaerak/kas_en.html  http://tilastokeskus.fi/meta/kas/kaupunki_maaseu_en.html | Official Statistics of Finland (OSF): Population structure [e-publication]. ISSN=1797-5395. Helsinki: Statistics Finland. |
| Annual change in population size |  |  |
| Females |  |  |
| <15-year-olds |  |  |
| ≥65-year-olds |  |  |
| Population density |  |  |
| Individuals living in rural area |  |  |
| Individuals living in municipality of birth |  |  |
| Foreign language speakers |  |  |
| Low education | http://www.stat.fi/til/vkour/meta_en.html  http://www.stat.fi/til/vkour/kas_en.html | Official Statistics of Finland (OSF): Educational structure of population [e-publication]. ISSN=2242-2919. Helsinki: Statistics Finland. |
| Unemployment | http://www.stat.fi/til/tyokay/meta_en.html  http://www.stat.fi/til/tyokay/kas_en.html | Official Statistics of Finland (OSF): Employment [e-publication]. ISSN=2323-6825. Helsinki: Statistics Finland. |
| Median annual income | http://www.stat.fi/til/tvt/meta_en.html  http://www.stat.fi/til/tvt/kas_en.html | Official Statistics of Finland (OSF): Taxable incomes [e-publication]. Helsinki: Statistics Finland. |
| Individuals per household unit  Overcrowded household units | http://www.stat.fi/til/asas/meta_en.html http://www.stat.fi/til/asas/kas_en.html | Official Statistics of Finland (OSF): Dwellings and housing conditions [e-publication]. ISSN=1798-6761. Helsinki: Statistics Finland. |
| Divorce rate | http://www.stat.fi/til/ssaaty/meta_en.html  https://www.stat.fi/til/ssaaty/kas_en.html | Official Statistics of Finland (OSF): Changes in marital status [e-publication]. ISSN=1797-643X. Helsinki: Statistics Finland. |
| Car ownership rate | http://www.stat.fi/til/mkan/meta_en.html  http://www.stat.fi/til/mkan/kas_en.html | Official Statistics of Finland (OSF): Motor vehicle stock [e-publication]. Helsinki: Statistics Finland. |
| Crime rate | http://www.stat.fi/til/rpk/meta_en.html  http://www.stat.fi/til/rpk/kas_en.html | Official Statistics of Finland (OSF): Statistics on offences and coercive measures [e-publication]. ISSN=2342-9178. Helsinki: Statistics Finland. |

All referred Oct 25, 2020.

**Supplementary Table 2.** Association between sociodemographic indicators and birth rate in Finland in 2011—2018.

| Indicator | Univariate models | | |  | Multivariable model #1 | | |  | Multivariable model #2 | | |  | Multivariable model #3 | | |  | Final multivariable model | | | |
| --- | --- | --- | --- | --- | --- | --- | --- | --- | --- | --- | --- | --- | --- | --- | --- | --- | --- | --- | --- | --- |
|  | RR | 95% CI | P |  | RR | 95% CI | P |  | RR | 95% CI | P |  | RR | 95% CI | P |  | RR | 95% CI | P |  |
| Population structure |  |  |  |  |  |  |  |  |  |  |  |  |  |  |  |  |  |  |  |  |
| Total population size | **1.06** | **1.02; 1.09** | **0.002** |  | **1.03** | **1.01; 1.04** | **0.003** |  | **1.02** | **1.01; 1.03** | **0.003** |  | 1.01 | 0.99; 1.03 | 0.530 |  | 1.00 | 0.98; 1.03 | 0.753 |  |
| Annual change in population size (%) | **1.09** | **1.08; 1.11** | **<0.001** |  | **1.05** | **1.03; 1.07** | **<0.001** |  | **1.06** | **1.05; 1.08** | **<0.001** |  | **1.06** | **1.05; 1.08** | **<0.001** |  | **1.06** | **1.04; 1.08** | **<0.001** |  |
| Females (%) | 1.02 | 0.98; 1.06 | 0.284 |  | 1.01 | 0.98; 1.03 | 0.625 |  | 1.02 | 0.99; 1.05 | 0.090 |  | 1.01 | 0.98; 1.04 | 0.405 |  | 1.01 | 0.99; 1.04 | 0.346 |  |
| <15-year-olds (%) | **1.30** | **1.27; 1.33** | **<0.001** |  | **1.23** | **1.18; 1.28** | **<0.001** |  | **1.18** | **1.14; 1.23** | **<0.001** |  | **1.27** | **1.20; 1.34** | **<0.001** |  | **1.29** | **1.22; 1.36** | **<0.001** |  |
| ≥65-year-olds (%) | **0.78** | **0.76; 0.80** | **<0.001** |  | 0.96 | 0.93; 1.00 | 0.071 |  | **0.89** | **0.84; 0.94** | **<0.001** |  | **0.89** | **0.84; 0.94** | **<0.001** |  | **0.90** | **0.85; 0.96** | **<0.001** |  |
| Education and income |  |  |  |  |  |  |  |  |  |  |  |  |  |  |  |  |  |  |  |  |
| Low education (%) | **0.87** | **0.84; 0.89** | **<0.001** |  | - |  |  |  | 0.99 | 0.96; 1.02 | 0.386 |  | 0.99 | 0.96; 1.02 | 0.538 |  | 0.97 | 0.94; 1.01 | 0.125 |  |
| Unemployment (%) | **0.94** | **0.91; 0.96** | **<0.001** |  | - |  |  |  | **0.97** | **0.95; 0.99** | **0.002** |  | **0.96** | **0.94; 0.99** | **0.001** |  | **0.98** | **0.95; 0.99** | **0.044** |  |
| Median annual income (eur) | **1.14** | **1.11; 1.17** | **<0.001** |  | **-** |  |  |  | **0.90** | **0.87; 0.92** | **<0.001** |  | **0.93** | **0.90; 0.97** | **<0.001** |  | **0.92** | **0.89; 0.96** | **<0.001** |  |
| Location and living |  |  |  |  |  |  |  |  |  |  |  |  |  |  |  |  |  |  |  |  |
| Population density (per km^2^) | **1.03** | **1.01; 1.06** | **0.008** |  | **-** |  |  |  | **-** |  |  |  | 1.01 | 0.99; 1.03 | 0.419 |  | 1.01 | 0.99; 1.03 | 0.495 |  |
| Individuals living in rural area (%) | **0.92** | **0.90; 0.95** | **<0.001** |  | - |  |  |  | - |  |  |  | 1.01 | 0.99; 1.03 | 0.334 |  | 1.01 | 0.99; 1.03 | 0.478 |  |
| Individuals per household unit | **1.22** | **0.19; 1.25** | **<0.001** |  | - |  |  |  | - |  |  |  | **0.93** | **0.89; 0.97** | **0.002** |  | **0.94** | **0.90; 0.98** | **0.004** |  |
| Overcrowded household units (%) | **1.14** | **1.11; 1.17** | **<0.001** |  | - |  |  |  | - |  |  |  | 0.99 | 0.96; 1.02 | 0.442 |  | 0.99 | 0.96; 1.02 | 0.488 |  |
| Individuals living in municipality of birth (%) | **0.93** | **0.90; 0.96** | **<0.001** |  | **-** |  |  |  | **-** |  |  |  | **1.05** | **1.03; 1.08** | **<0.001** |  | **1.05** | **1.03; 1.08** | **<0.001** |  |
| Other indicators |  |  |  |  |  |  |  |  |  |  |  |  |  |  |  |  |  |  |  |  |
| Foreign language speakers (%) | 1.02 | 0.99; 1.05 | 0.103 |  | - |  |  |  | - |  |  |  | - |  |  |  | **1.02** | **1.01; 1.04** | **0.019** |  |
| Divorce rate | 1.00 | 0.99; 1.01 | 0.570 |  | - |  |  |  | - |  |  |  | - |  |  |  | 1.00 | 0.99; 1.02 | 0.807 |  |
| Car ownership rate | **0.95** | **0.91; 0.98** | **0.004** |  | - |  |  |  | - |  |  |  | - |  |  |  | 1.02 | 0.99; 1.04 | 0.096 |  |
| Crime rate | 1.00 | 0.98; 1.02 | 0.995 |  | - |  |  |  | - |  |  |  | - |  |  |  | 1.00 | 0.99; 1.01 | 0.872 |  |

Univariate, partially adjusted, and final multivariable models. Predictors were standardized before fitting the GEE models, making results harmonized into standard deviation units.
CI = Confidence interval, GEE = Generalized estimating equations, RR = Rate ratio.

**Supplementary Table 3**. Intercorrelations of the predictor variables.

| Variable | Total pop | | Change in pop | | Females | | <15-y-os | | ≥65-y-os | | Low educ | | Unempl | | Income | | Pop density | | Rurality | | Household size | | Overcrowding | | Birth municip | | Foreign lang | | Divorces | | Cars | | Crimes | |
| --- | --- | --- | --- | --- | --- | --- | --- | --- | --- | --- | --- | --- | --- | --- | --- | --- | --- | --- | --- | --- | --- | --- | --- | --- | --- | --- | --- | --- | --- | --- | --- | --- | --- | --- |
|  | R | P | R | P | R | P | R | P | R | P | R | P | R | P | R | P | R | P | R | P | R | P | R | P | R | P | R | P | R | P | R | P | R | P |
| Total population size | **1** |  |  |  |  |  |  |  |  |  |  |  |  |  |  |  |  |  |  |  |  |  |  |  |  |  |  |  |  |  |  |  |  |  |
| Annual change in population size (%) | 0.288 | <0.001 | **1** |  |  |  |  |  |  |  |  |  |  |  |  |  |  |  |  |  |  |  |  |  |  |  |  |  |  |  |  |  |  |  |
| Females (%) | 0.371 | <0.001 | 0.288 | <0.001 | **1** |  |  |  |  |  |  |  |  |  |  |  |  |  |  |  |  |  |  |  |  |  |  |  |  |  |  |  |  |  |
| <15-year-olds (%) | 0.036 | 0.074 | 0.511 | <0.001 | 0.118 | <0.001 | **1** |  |  |  |  |  |  |  |  |  |  |  |  |  |  |  |  |  |  |  |  |  |  |  |  |  |  |  |
| ≥65-year-olds (%) | -0.295 | <0.001 | -0.659 | <0.001 | -0.294 | <0.001 | -0.829 | <0.001 | **1** |  |  |  |  |  |  |  |  |  |  |  |  |  |  |  |  |  |  |  |  |  |  |  |  |  |
| Low education (%) | -0.353 | <0.001 | -0.451 | <0.001 | -0.393 | <0.001 | -0.428 | <0.001 | 0.528 | <0.001 | **1** |  |  |  |  |  |  |  |  |  |  |  |  |  |  |  |  |  |  |  |  |  |  |  |
| Unemployment (%) | 0.015 | 0.447 | -0.330 | <0.001 | -0.051 | 0.011 | -0.332 | <0.001 | 0.350 | <0.001 | 0.109 | <0.001 | **1** |  |  |  |  |  |  |  |  |  |  |  |  |  |  |  |  |  |  |  |  |  |
| Median annual income (eur) | 0.259 | <0.001 | 0.546 | <0.001 | 0.338 | <0.001 | 0.464 | <0.001 | -0.581 | <0.001 | -0.690 | <0.001 | -0.527 | <0.001 | **1** |  |  |  |  |  |  |  |  |  |  |  |  |  |  |  |  |  |  |  |
| Population density (per km^2^) | 0.742 | <0.001 | 0.267 | <0.001 | 0.325 | <0.001 | 0.037 | 0.065 | -0.245 | <0.001 | -0.281 | <0.001 | -0.100 | <0.001 | 0.354 | <0.001 | **1** |  |  |  |  |  |  |  |  |  |  |  |  |  |  |  |  |  |
| Individuals living in rural area (%) | -0.446 | <0.001 | -0.429 | <0.001 | -0.495 | <0.001 | -0.276 | <0.001 | 0.507 | <0.001 | 0.636 | <0.001 | 0.081 | <0.001 | -0.578 | <0.001 | -0.371 | <0.001 | **1** |  |  |  |  |  |  |  |  |  |  |  |  |  |  |  |
| Individuals per household unit | -0.129 | <0.001 | 0.394 | <0.001 | -0.124 | <0.001 | 0.891 | <0.001 | -0.688 | <0.001 | -0.210 | <0.001 | -0.410 | <0.001 | 0.331 | <0.001 | -0.058 | 0.004 | -0.086 | <0.001 | **1** |  |  |  |  |  |  |  |  |  |  |  |  |  |
| Overcrowded household units (%) | -0.021 | 0.294 | 0.229 | <0.001 | -0.221 | <0.001 | 0.672 | <0.001 | -0.481 | <0.001 | 0.032 | 0.108 | -0.117 | <0.001 | -0.012 | 0.563 | 0.000 | 0.985 | 0.115 | <0.001 | 0.706 | <0.001 | **1** |  |  |  |  |  |  |  |  |  |  |  |
| Individuals living in municipality of birth (%) | -0.187 | <0.001 | -0.488 | <0.001 | -0.349 | <0.001 | -0.344 | 0.001 | 0.497 | <0.001 | 0.504 | <0.001 | 0.381 | <0.001 | -0.774 | <0.001 | -0.327 | <0.001 | 0.566 | <0.001 | -0.211 | <0.001 | 0.009 | 0.644 | **1** |  |  |  |  |  |  |  |  |  |
| Foreign language speakers (%) | 0.529 | <0.001 | 0.269 | <0.001 | 0.257 | <0.001 | -0.087 | <0.001 | -0.154 | <0.001 | -0.174 | <0.001 | -0.286 | <0.001 | 0.430 | <0.001 | 0.549 | <0.001 | -0.325 | <0.001 | -0.169 | <0.001 | -0.161 | <0.001 | -0.371 | <0.001 | **1** |  |  |  |  |  |  |  |
| Divorce rate | 0.224 | <0.001 | 0.198 | <0.001 | 0.409 | <0.001 | 0.126 | <0.001 | -0.270 | <0.001 | -0.300 | <0.001 | 0.031 | <0.001 | 0.289 | <0.001 | 0.211 | <0.001 | -0.380 | <0.001 | -0.068 | <0.001 | -0.086 | <0.001 | -0.346 | <0.001 | 0.137 | <0.001 | **1** |  |  |  |  |  |
| Car ownership rate | -0.396 | <0.001 | -0.170 | <0.001 | -0.343 | <0.001 | -0.259 | <0.001 | 0.316 | <0.001 | 0.369 | <0.001 | -0.408 | <0.001 | 0.033 | 0.095 | -0.298 | <0.001 | 0.422 | <0.001 | -0.097 | <0.001 | -0.173 | <0.001 | 0.027 | 0.181 | 0.075 | <0.001 | -0.219 | <0.001 | **1** |  |  |  |
| Crime rate | 0.029 | 0.142 | 0.036 | 0.071 | 0.117 | <0.001 | 0.019 | 0.342 | -0.060 | 0.003 | -0.048 | <0.001 | 0.025 | 0.212 | 0.015 | 0.447 | -0.001 | 0.958 | 0.001 | 0.957 | -0.057 | 0.005 | -0.071 | <0.001 | -0.072 | <0.001 | 0.007 | 0.733 | 0.095 | <0.001 | -0.087 | <0.001 | **1** |  |

P = P value, R = Pearson’s correlation coefficient.
